# Ferulic Acid: A Natural Phenol That Inhibits Neoplastic Events through Modulation of Oncogenic Signaling

**DOI:** 10.3390/molecules27217653

**Published:** 2022-11-07

**Authors:** Hardeep Singh Tuli, Ajay Kumar, Seema Ramniwas, Renuka Coudhary, Diwakar Aggarwal, Manoj Kumar, Ujjawal Sharma, Nidarshana Chaturvedi Parashar, Shafiul Haque, Katrin Sak

**Affiliations:** 1 Department of Biotechnology, Maharishi Markandeshwar Engineering College, Maharishi Markandeshwar (Deemed to be University), Mullana-Ambala 133207, India; 2 Punjab Biotechnology Incubator (PBTI), Phase VIII, Mohali 160071, India; 3 University Centre for Research and Development, University Institute of Pharmaceutical Sciences, Chandigarh University, Gharuan, Mohali 140413, India; 4 Department of Chemistry, Maharishi Markandeshwar University, Sadopur-Ambala 134007, India; 5 Department of Human Genetics and Molecular Medicine, Central University of Punjab, Bhatinda 151001, India; 6 Research and Scientific Studies Unit, College of Nursing and Allied Health Sciences, Jazan University, Jazan 45142, Saudi Arabia; 7 NGO Praeventio, 50407 Tartu, Estonia

**Keywords:** ferulic acid, apoptosis and cell cycle arrest, anti-angiogenesis, anti-metastasis, synergism

## Abstract

Despite the immense therapeutic advances in the field of health sciences, cancer is still to be found among the global leading causes of morbidity and mortality. Ethnomedicinally, natural bioactive compounds isolated from various plant sources have been used for the treatment of several cancer types and have gained notable attention. Ferulic acid, a natural compound derived from various seeds, nuts, leaves, and fruits, exhibits a variety of pharmacological effects in cancer, including its proapoptotic, cell-cycle-arresting, anti-metastatic, and anti-inflammatory activities. This review study presents a thorough overview of the molecular targets and cellular signaling pathways modulated by ferulic acid in diverse malignancies, showing high potential for this phenolic acid to be developed as a candidate agent for novel anticancer therapeutics. In addition, current investigations to develop promising synergistic formulations are also discussed.

## 1. Introduction

Over the past few decades, it has become more and more popular to study the role of natural plant-derived compounds in a wide range of models for chronic diseases, especially against different types of human cancers [1]. One reason for this is the continuously raising incidence of these aging-related disorders all over the world [2]. On the other hand, as there are no curative treatment options frequently available, findings regarding new safe and efficient therapeutics are increasingly genuine. It is indeed well known that plants are able to synthesize a large spectrum of structurally unrelated molecules, many of which have been demonstrated to reveal diverse bioactivities in human experimental systems [3]. As a result, some specific compounds, such as alkaloids vincristine and vinblastine and terpenoid paclitaxel, have been developed in chemotherapeutics, commonly used in the clinical settings today, whereas several others are currently under clinical trials as adjunctive therapies against different cancer types [3].

The most widespread class of compounds among the huge diversity of phytochemicals constitutes phenolic agents, further divided into polyphenols with flavonoids and tannins, and simple phenols, including phenolic acids [4]. Ferulic acid is a hydroxycinnamic acid ubiquitously occurring in the plant kingdom and is derived from various vegetable sources, such as leaves, fruits, seeds, and nuts [5]. A number of studies have described the diverse bioactivities of this phenolic acid, and especially its anticancer potential. This natural compound can exert anti-inflammatory, antiproliferative, proapoptotic, antiangiogenic, and/or antimetastatic effects in various experimental models of malignancies, including lung cancer [6], colorectal cancer [7], liver cancer [8], breast cancer [9], cervical cancer [10], osteosarcoma [11] and glioblastoma [12]. In doing so, ferulic acid is able to attack multiple molecular targets and alter several cellular signaling pathways, which ultimately results in the inhibition of malignant development and tumor growth [13].

In this review article, the current knowledge regarding the anticancer potential of ferulic acid is compiled and systematically presented, and it is then analyzed in addition to the role of this phytochemical on the resistance mechanisms of conventional chemotherapeutic drugs. Moreover, the co-effects of ferulic acid, along with other therapeutic modalities, are discussed, presenting synergistic combinations that are most valuable for further studies. Considering the generally low bioavailability characteristic for natural phenolics, nanotechnological possibilities to improve the targeted delivery of ferulic acid to the tumoral sites are discussed. In this way, relying on the complex picture, further steps can be readily planned for applications of the anticancer properties of ferulic acid in the fight against cancer.

## 2. Sources, Chemistry, and Structural Activity Relationship of Ferulic Acid

Chemically, ferulic acid is 4-hydroxy-3-methoxycinnamic acid or (*E*)-3-(4-hydroxy-3-methoxyphenyl) acrylic acid, occurring in two isomeric forms, i.e., *cis* and *trans*. It is found naturally in various plants such as citrus fruits, wheat, spinach, sugar beets, cereals, sugarcane bagasse, neem, and pineapples [14]. It has also been reported in Chinese medicine herbs, including *Angelica sinensis*, *Cimicifuga heracleifolia*, and *Lignsticum chuangxiong*. Ferulic acid can be synthesized in a laboratory through the condensation of vanillin with malonic acid in the presence of piperidine. However, this reaction takes three weeks to complete, but the yield is found to be high with a mixture of *trans*- and *cis*-isomers. Nevertheless, Da and Xu successfully reduced this reaction time to 2 h by utilizing benzylamine as a catalyst and methylbenzene as the solvent at 85–95 °C [15].

**Figure 1:**
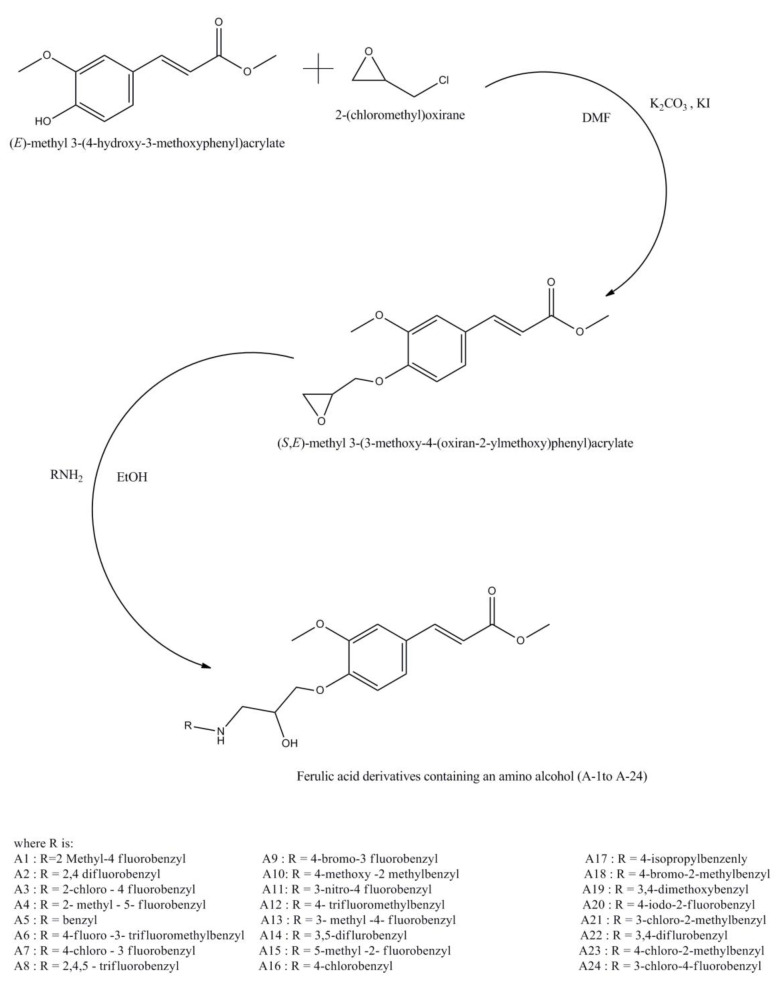
Chemical structure of ferulic acid and synthetic routes of its derivatives.

It has been reported that the biological activity of ferulic acid can be altered by creating its derivatives. A series of such derivatives of ferulic acid with β-amino alcohol has been previously reported (Figure 1) [16]. The chemical structure of ferulic acid has the presence of benzene rings with a carboxylic group. Hydroxyl groups present at C1 and C9 are considered to be the main sources of antioxidant character. The double bond presented between C2-C3 is known to be responsible for effective bio-activity [17]. Furthermore, the carboxylic group of ferulic acid provides an easily made ester, which is in turn responsible for cholesterol-lowering activity [15].

## 3. In Vivo Pharmacokinetics of Ferulic Acid

In rodents and humans, the absorption and metabolism of ferulic acid has been widely studied and reported. Polyphenolic compounds are mostly consumed as simple phenolic acids. It has been reported that, in the stomach, the rate of absorption of ferulic acid is relatively faster than other phenolic compounds, and that it can be absorbed along the entire gastrointestinal tract [18]. The metabolism of ferulic acid mainly occurs in the liver, and forms conjugated products with glucuronides, sulfate, and sulfoglucuronide [19]. In humans, ferulic acid is excreted in urine as 3-hydroxyphenyl and 3-methoxy-4-hydroxy phenyl derivatives of phenyl propionic acid, hydracrylic acid, and glycine conjugates after metabolism. In rats, ferulic acid itself is partly excreted as glucuronide, as revealed in feeding studies. However, 3-hydroxy phenylpropionic acid is excreted by rats as a major urinary metabolite after the intraperitoneal administration of ferulic acid [20]. After a single administration, the distribution of ferulic acid in the body is substantial. For example, distribution of ferulic acid is ~4% in the gastric mucosa after oral administration; it is 10% in the blood pool, kidney, and liver, and distribution in other tissues is 53% [18]. Due to the low toxicity of ferulic acid, it has been reported to be a relatively safer molecule. The LD_50_ value is lower for female rats (2113 mg kg^−1^ body weight) in comparison to male rats (2445 mg kg^−1^ body weight) [21].

## 4. Cellular Mechanism of Ferulic Acid in Cancer

### 4.1. Induction of Apoptosis and Cell Cycle Arrest

**Figure 2:**
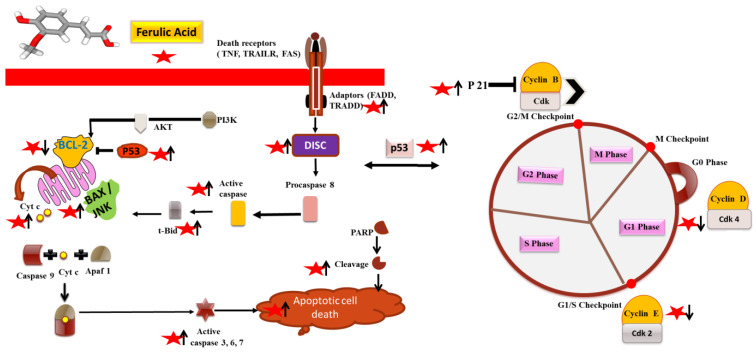
Molecular targets of ferulic acid in signaling processes leading to cell cycle arrest and apoptosis. Bcl-2-associated X protein (BAX), BCL-2 antagonist/killer (BAK), tumor suppressor p53 (p53) and cytochrome complex (CYCS), B-cell lymphoma 2 (Bcl-2), Poly (ADP-ribose) polymerase (PARP), Fas-ligand (FASL), and TNFR-associated death domain (TRADD).

Programed cell death, apoptosis, is one of the most preferred target pathways for inhibition of proliferation and growth of the tumor by anticancer therapies. Chemo-preventive phytochemicals are known to mediate apoptosis either via intrinsic (mitochondrial) or extrinsic mechanisms (death receptor). It has been discovered that these compounds upregulate apoptotic protein and downregulate anti-apoptotic protein (Figure 2). For instance, by upregulating the tumor suppressor and apoptotic genes Bcl-2-associated X protein (BAX), BCL-2 interacting killer (BIK), tumor suppressor p53 (p53), and cytochrome complex (CYCS) and downregulating the expression of the antiapoptotic protein B-cell lymphoma 2 (Bcl-2), ferulic acid causes apoptosis in prostate cancer cells [22]. Furthermore, Luo et al. observed that reduced levels of Myeloid cell leukemia 1 (Mcl-1) and Bcl-2, and increased Bax levels after ferulic acid treatment resulted in apoptosis in human cervical cancer cell lines (HeLa and Caski) [23]. Caspase (CASP)-8, Fas-ligand (FASL), and Poly (ADP-ribose) polymerase (PARP) are three extrinsic mechanisms of apoptotic cell death that are associated with the expression of molecular proteins. According to Kampa et al., FAS/FASLG caused the human breast cancer cell line (T47D) to undergo apoptosis [24]. Ferulic acid treatment leads to the induction of apoptosis via elevated expressions of the apoptotic proteins CASP1, CASP2, CASP8, FASLG, FAS, and TNFR-associated death domain (TRADD) in the prostate cancer cell line (LNCaP) [25]. Through regulation of p53, Bax, caspase-3, and growth arrest and DNA-damage (GADD45), treatment with ferulic acid has been shown to begin apoptosis in non-small cell lung cancer cells (NCI-H460) [26]. According to Grasso et al., free ferulic acid significantly reduced the levels of Bcl-2 and Master Regulator of Cell Cycle proteins (c-Myc) expression along with caspase-3 and PARP-1 cleavage, which activated the apoptotic pathway [27]. DNA fragmentation, which is the hallmark of apoptosis, was determined in Caski cells after ferulic acid treatment [28]. Ferulic acid reduced the phosphorylation of protein kinase B (Akt) and Phosphoinositide 3-kinase (PI3K) in Caski cells. In osteosarcoma cells, ferulic acid augmented the Bax expression, decreased the Bcl-2 expression, and then increased the activity of caspase-3, and induced death by blocking the PI3K/Akt pathway [28].

Cyclins (CCN), cyclin-dependent kinase (CDKI) inhibitors, and cyclin-dependent kinases (CDK) are known to arrest cell cycle progression. By upregulating CDKN1A (P21) protein expression and downregulating CCND1 and phosphorylated retinoblastoma protein (RB) levels in the endothelial cells (ECV304), ferulic acid produced cell cycle arrest in the G0/G1 phase [29]. Ferulic acid treatment inhibited PI3K/Akt pathway and induced G0/G1 phase arrest via downregulation of expression of cell cycle-related proteins CDK2, CDK4, and CDK6 in osteosarcoma cells [30]. In HeLa and Caski cells, Gao et al. demonstrated that ferulic acid caused cell death and G0/G1 phase arrest by increasing the cell cycle-related proteins, such as p53 and p21 expression, and by lowering CCND1 and CCNE levels [31]. According to Janick et al., ferulic acid led to an arrest of the cell cycle in the S phase and had an antiproliferative effect on colon cancer cells (Caco-2) [32]. Due to the decreased expression of genes that were crucial for cell cycle arrest in the G1/S phase in prostate cancer cells, ferulic acid may prevent cell cycle progression. The Transcription Factor 4 (E2F4) gene expression that was much higher in the ferulic acid-treated cells caused arrest of the cell cycle at the G0/G1 stages in prostate cancer cells (PC-3) due to downregulation of transcription by creating a complex with RB1 [25].

In addition, ferulic acid is also known to induce autophagy in cancer cells. It is a natural breakdown of the cell to eliminate malfunctioning components through a lysosome-dependent controlled mechanism. For instance, using hepatocellular carcinoma (HepG2) cells, Wang et al. in 2022 determined that proliferative ability was decreased by ferulic acid by elevating the levels of the apoptosis and autophagy biomarkers, including beclin-1, Light chain (LC3-I/LC3-II), PTEN-induced putative kinase 1 (PINK-1), and Parkin [33]. Similar to this, utilizing the ferulic acid derivative tributyltin(IV) ferulate (TBT-F) on colon cancer cells (HCT116, HT-29 and Caco-2) led to an increase in autophagy-related proteins, such as LC3-II, and receptors of autophagy (p62) [34]. Therefore, genes or proteins involved in apoptosis and cell cycle regulation are significant in the development of anticancer drugs, and research on these genes for cancer therapies has been constantly growing.

### 4.2. Antiangiogenic Action of Ferulic Acid

There are two main hallmarks of cancer progression: uninhibited angiogenesis and development of vascular architecture [35]. Angiogenesis is the process of creating new blood vessels from previous ones and is essential for transporting oxygen, nutrients, and growth hormones to distant organs for the development of cells, playing an important role in cancer progression [36]. Through the release of chemical signals that promote angiogenesis, tumors maintain blood supply by regulating both positive (angiogenic) and negative (anti-angiogenic) endogenous factors, such as adult endothelial cells (ECs). Angiogenesis is crucial for the development of numerous illnesses, as well as for regular physiological processes like the formation of an embryo, the healing of wounds, and the menstrual cycle. It is generally recognized that angiogenesis is dysregulated in a number of diseases, including psoriasis, diabetic retinopathy, malignant tumors, rheumatoid arthritis, and age-related macular degeneration (AMD). An unbalance between numerous pro-angiogenic and anti-angiogenic factors is important for angiogenesis [37]. Angiogenesis process is initiated by pro-angiogenic signals, inflammation, ischemia, hypoxia, and other variables that act on cytokines, as well as angiogenic factors like vascular endothelial growth factor (VEGF) or fibroblast growth factor (FGF) in tumor cells, urokinase-type plasminogen activator (uPA), and adrenomedullin (ADM) [38]. As a result, stopping angiogenesis is an effective therapeutic strategy for the management of a number of illnesses, including cancer. Despite being widely available, anti-angiogenic medications like bevacizumab, pegaptanib, ranibizumab, sunitinib, sorafenib, regorafenib, and axitinib also have serious side effects, such as cardiovascular toxicity, bleeding risk, intraocular inflammation, ocular hemorrhage, and retinal detachment [39]. In order to complement and integrate with current therapies, innovative and efficient treatments that precisely target angiogenesis and have fewer side effects need to be researched, developed, and tested [40]. Among the broad spectrum of botanicals, ferulic acid is one of the potent constituents found in many vegetables and has numerous pharmacological activities, such as anti-cancer, anti-inflammation, neuroprotective, anti-coagulation, and anti-angiogenesis [41]. In a normal cellular microenvironment using human umbilical vein endothelial cells (HUVECs), Lin et al. [42] determined that ferulic acid significantly augmented angiogenesis by increasing VEGF, platelet-derived growth factor (PDGF), and hypoxic-induced factor (HIF) 1α expression via mitogen-activated protein kinase and PI3K pathways. Whereas, Yang et al., 2015 [43], reported that ferulic acid reduced the growth of melanoma cells (A375, CHL-1 and SK-MEL-2) via suppressing FGF1, leading to the activation of FGFR1 and PI3K-Akt signaling. In addition, ferulic acid showed anticancer potential by suppressing angiogenesis and causing inhibition of melanoma growth in a xenograft model [44]. Researchers have reported that ferulic acid significantly revealed antiangiogenic properties in a chorioallantoic membrane (CAM) model of chicken eggs through downregulation of VEGF-2 and cyclooxygenase (COX-2) expression. Therefore, anti-angiogenic mechanisms can be considered promising for the future design of novel therapeutics.

### 4.3. Inhibition of Metastasis and Invasion

**Figure 3:**
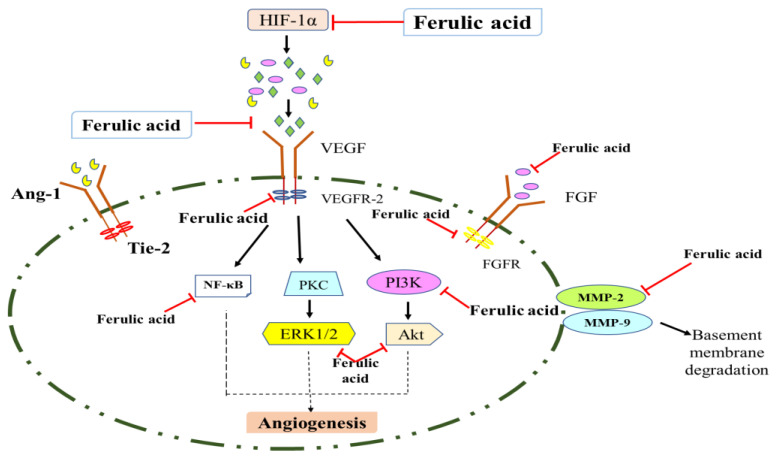
Major signaling pathways targeted by ferulic acid in angiogenesis and metastasis processes. Vascular endothelial growth factor (VEGF), Angiopoietin-1 (Ang1), Tyrosine-protein kinase (Tie-2), Hypoxic-inducible factor (HIF) 1α, Protein kinase B (Akt), Phosphoinositide 3-kinase (PI3K), protein kinase (PKC), Nuclear factor kappa light chain enhancer of activated B cells (NF-κB), Extracellular signal-regulated kinase (ERK), Matrix metalloproteinases (MMPs).

Another main hallmark of malignant tumors is believed to be invasion and metastasis that lead to clinical death [45]. Tumor invasion and metastasis mechanisms involve the detachment of cancer cells from the main tumor, migration, angiogenesis, and proliferation to distant tissues [46]. Beyond the boundaries of the healthy tissue from which they originate, cancer cells can enter the bloodstream, travel to distant organs, and ultimately cause the development of secondary tumors, known as metastases. Matrix metalloproteinases (MMPs) play a significant role in the growth of malignancies by disrupting natural invasion barriers [47]. The zinc-dependent endopeptidases MMP-2 and MMP-9 are associated with tumor invasion and metastasis due to their capacity to remodel tissue by degrading the basement membrane and extracellular matrix, thereby triggering angiogenesis (Figure 3) [48]. Therefore, the largest problem in cancer chemotherapy has been preventing the phenomenon of invasion and metastasis. Many natural constituents such as polyphenols, terpenoids, flavonoids, alkaloids, steroids, and saponins have the potential to be anti-invasive and anti-metastatic. Zhang et, 2016 [49], reported that ferulic acid showed antimetastatic potential against breast cancer cells (MDA-MB-231) by upregulating caspase-3 and downregulating epithelial-mesenchymal transition (EMT). Ferulic acid oral dose significantly reduced the tumor volume in MDA-MB-231 xenografts in female BALB/c nude mice, and showed no toxicity at a dose of 100 mg/kg body weight of animals. El-Gogary et al., 2022 [50], investigated that nanoencapsulated ferulic acid exhibited anticancer potential in colorectal cancer cell lines (HCT-116 and Caco2), and ferulic acid lipid encapsulated nanoparticles showed significant antioxidant, apoptotic, anti-angiogenic, and anti-inflammatory properties in vivo through downregulation of cyclin D1, Insulin-like growth factor (IGF II), and VEGF and modulation of BAX/Bcl-2. Therefore, inhibition of cancer migration from one site to another can significantly increase patients’ survival rates, and researchers are currently exploring anti-metastatic drugs from plant origins.

### 4.4. Anti-Inflammatory Mechanisms

**Figure 4:**
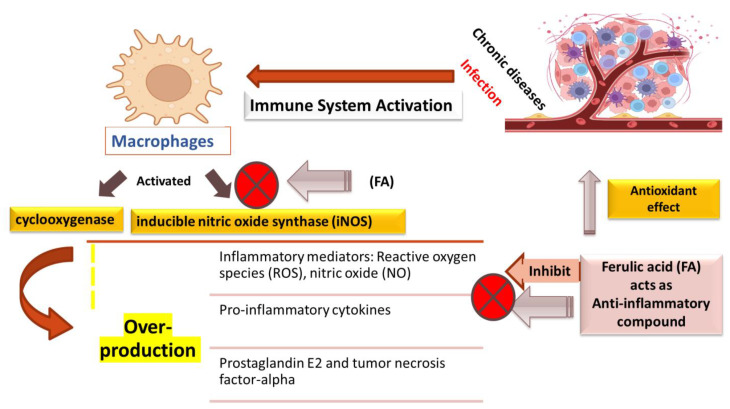
Anti-inflammatory targets of ferulic acid.

For the emergence and spread of chronic illnesses, inflammatory and immune responses act as key regulators. Activated inflammatory cells mediate chronic and acute inflammation through a multi-step process [41]. In several in vitro and in vivo models, it has been reported that ferulic acid possesses anti-inflammatory action. In vitro inflammation is widely studied in LPS-treated murine macrophages (Raw 264.7) [19] The overproduction of inflammatory mediators (reactive oxygen species (ROS), nitric oxide (NO), pro-inflammatory cytokines, and prostaglandin E2 (PGE2)) generated by activated inducible nitric oxide synthase (iNOS) and COX is centrally managed by the macrophages generated by the immune system [51]. Ferulic acid acts as an antioxidant and decreases macrophage inflammatory protein-2 (MIP-2) production [52]. Ferulic acid and its derivatives also inhibit the expression of inflammatory mediators, such as iNOS, NO production, prostaglandin E2, and tumor necrosis factor-alpha (TNFα) in cells stimulated by the bacterial endotoxin lipopolysaccharide [53,54,55]. A recent study showed that ferulic acid isolated from corn also inhibited the iNOS expression and NO production in lipopolysaccharide (LPS)-stimulated Raw 264.7 cells [56]. In addition to this, it has been reported that ferulic acid derivative feruloyl-myo-inositol leads to the suppression of cyclooxygenase-2 promoter activity in human colon cancer (DLD-1) cells via β-galactosidase reporter gene assay system [57]. In a concentration-dependent manner, ferulic acid leads to the inhibition of chemokine superfamily member (murine MIP-2) as studied in LPS-stimulated macrophages (RAW 264.7) cells. The anti-inflammatory response of ferulic acid (20 mg/kg) was studied in vivo also in rats, showing reduction of the expression of cyclooxygenase-2 and nuclear factor kappa light chain enhancer of activated B cells (NF-κB) in lung and liver, which was increased by nicotine treatment [58]. These findings suggest that ferulic acid has anti-inflammatory mechanisms against inflammatory diseases (Figure 4).

## 5. Synergistic Interactions of Ferulic Acid in Cancer

The use of diverse synergistic chemo-preventive techniques with improved sensitivity in combination with known chemotherapeutic medications has received a lot of attention. Evidence has suggested that synergisms provide maximum therapeutic efficacy, minimal side effects, and overcome drug resistance [59]. When given in combination with δ-tocotrienol, ferulic acid was reported to synergistically inhibit telomerase activity in human colorectal adenocarcinoma cells (DLD-1) by synergistically down-regulating the expression of human telomerase reverse transcriptase (hTERT), the catalytic subunit of the enzyme, suggesting that ferulic acid might augment the anti-cancer activity of δ-T3 [60]. Additionally, the combination of δ-tocotrienol and ferulic acid has also been investigated, showing synergistic inhibitory effects and preventing the spread of different forms of cancer cells, including prostate cancer (DU-145), breast cancer (MCF-7), and pancreatic cancer (PANC-1) cells. Synergistic therapy has been found to be more effective and remarkably reduced cell proliferation, as compared to δ-tocotrienol and ferulic acid alone [61]. Moreover, the compound has also been demonstrated to exert anti-proliferative/pro-apoptotic effects and to decrease the metastatic or angiogenic properties of different cancer cells when given in combination with unsaturated tocotrienols (TTs) [62]. Furthermore, recent studies have shown the concerted pro-apoptotic effects of ferulic acid and nanostructured lipid carrier in glioblastoma cells, thus increasing its bioavailability in the glioblastoma cells by escalating the effects on protein expression levels and on the activation of the apoptotic pathway more conspicuously when the cells were exposed to ferulic acid loaded in nanostructured lipid carriers (NLCs), as compared to free ferulic acid [63]. In addition, the combinatorial therapy of ferulic acid and cisplatin has also been reported to synergistically inhibit cellular proliferation in human leukemia cancer cell lines, and the synergized growth inhibitory effect with cisplatin was shown to be probably associated with the G2/M arrest in the cell cycle progression, thus indicating ferulic acid to be a better modulating agent on human malignant cell lines [64]. Furthermore, the combined effect of ferulic acid and gemcitabine on apoptosis and metastasis was also investigated, and the expression of various genes involved in apoptosis and metastasis was found to be increased with a higher fold change compared to the single treatment of gemcitabine in human prostate cancer cell (PC-3) lines [65]. Additionally, ferulic acid in combination with PARP inhibitors has been reported to sensitize breast cancer cells, thus serving as an effective combination chemotherapeutic agent as a natural bioactive compound [66]. Furthermore, the potential role of a combination formula of ferulic acid and aspirin was also explored for pancreatic cancer chemoprevention, utilizing a new chitosan-coated solid lipid nanoparticles (c-SLN) drug delivery system encapsulating the natural compound and the drug; the results were found to be promising [67]. Moreover, a study including two different polyphenols, curcumin, and ferulic acid as adjuvant chemotherapeutics was carried out, evaluating chemoresistance and cisplatin-induced ototoxicity against chemotherapeutic regimes, such as cisplatin, for different types of cancers. The use of adjuvants was found to be an effective tool for cancer therapy targeting ROS-modulated pathways [68]. Furthermore, a combination of caffeic and ferulic acid lipophilic derivatives also showed increased cytotoxicity toward human breast cancer cell lines, and thus could be applicable for chemopreventive and/or chemotherapeutic purposes [69]. From the above discussion, it can be determined that this bioactive natural substance has the potential to have synergistic effects on growth inhibition, apoptosis induction, and anticarcinogenic properties, and it may prove to be a promising alternative approach for boosting therapeutic potency and lowering systemic toxicity of chemotherapeutic drugs [70].

## 6. Safety Studies

As a natural plant-derived compound, ferulic acid is considered to be generally safe. Although several investigations have confirmed this assumption, systematic studies on its safety are still required before the development of ferulic acid as a therapeutic tool [71]. Actually, trans-ferulic acid-4-β-glucoside revealed no apparent toxicity in mice models, inducing no significant alterations in the body weight and blood biochemical parameters of animals [72]. Additionally, topical applications of ferulic acid did not cause any skin irritation in nude mice, representing an efficient and safe route for using ferulic acid against photodamage [73]. However, a long-term treatment with ferulic acid was still demonstrated to cause some nephrodamaging effects in the model of doxorubicin-induced chronic kidney disease in rats [74], suggesting that further studies on the safety of this phenolic acid are needed to determine the values of no-observed-adverse-effect-level (NOAEL) in health risk assessments. Table 1 and Table 2 represent an overview of ferulic acid anticancer effects.

## 7. Conclusions and Future Perspectives

The evidence presented in this review study clearly suggests that ferulic acid can be considered a possible option for the development of novel anticancer agents due to its capacity to disrupt cancer cell signaling. Several studies have reported the anti-neoplastic role of ferulic acid in various cancer cells, including brain cancer, breast cancer, gastric cancer, prostate cancer, cervical cancer, and colorectal cancer. Together, it can be established that this bioactive natural substance may have effects on tumor growth inhibition, apoptosis induction, suppression of angiogenesis, and metastasis, and may prove to be one of the most promising alternatives to current chemotherapeutic treatment methods for increasing therapeutic potency and lowering systemic toxicity. However, ferulic acid stability and limited solubility in aqueous media continue to be key obstacles to its bioavailability, preclinical efficacy, and clinical use. In this context, ferulic acid-loaded nano-therapeutic strategies, such as ionic gelation methods, can play an important role in overcoming these problems. For instance, chitosan-based nano-formulations can improve the stability of ferulic acid by modulating the hydrophobic interactions. Furthermore, investigations on synergistic combinations between ferulic acid and conventional anticancer drugs must be continued to find a more efficient dosage regimen for the treatment of diverse types of malignancies, inducing lower adverse side effects. Overall, ferulic acid presents a promising natural agent for supplementing the current anticancer arsenal with improved life expectancy and quality of life for patients.

## Figures and Tables

**Table 1:** Anticancer effects of ferulic acid based on in vitro studies.

Type of Cancer	Cell Lines	Effects	Mechanisms	Concentration	References
Melanoma	Murine B16	-	↓ melanin production, ↓ tyrosinase activity, ↓ casein kinase 2 (CK2), ↑ p- tyrosinase	25 and 50 µM	[75]
	A375, CHL-1, SK-MEL-2, B16F10	Anti-angiogenic	↓ proliferation, migration and tube formation, ↓ fibroblast growth factor 1 (FGF1), ↓ FGFR1, ↓ PI3K, ↓ protein kinase B (Akt) signaling, ↓ PI3K-Akt pathway, ↑ (HUVEC) Growth, ↓ VEGF-A, FGF1, FGF2, PDGF-α, PDGF-β and phosphatidylinositol-glycan biosynthesis class f protein (PIGF)	0, 2.5, 5, 10, 20, 30, 40 μM	[43]
Sarcoma	S180	Ameliorating oxidative stress injury	↓ diosbulbin B-induced liver injury, ↓ ALT and AST activities, Ferulic acid reverses diosbulbin B-decreased CuZn-SOD and CAT enzymatic activities and mRNA expression	-	[76]
Osteosarcoma	143B and MG63	Induces apoptosis	↓ proliferation, ↑ G0/G1 phase arrest, ↓ CDK 2, CDK 4, CDK 6, ↑ Bax, ↓ Bcl-2, ↑ caspase-3 activity, ↓ PI3K/Akt activation	0,10,30,100 and 150 µM	[77]
Thyroid	TT cells	Induces apoptosis	↓ invasion, migration and colony formation, ↓ URG4/URGCP (upregulated gene-4/upregulator of cell proliferation), ↓ CCND1, CDK4, CDK6, BCL2, MMP2, and MMP9, ↑ p53, PARP, PUMA, NOXA, BAX, BID, CASP3, CASP9 and TIMP1	50, 75, 100, 150, 200, 300, 400, 500, 750 μM and 1 mM	[78]
Breast	MDA-MB 231	Induces apoptosis	↓ proliferation, ↑ apoptotic cells, ↓ percentages of cells in G0/G1 phases by TQ, ↓ in %ages of cells in the S phase by FA	Thymoquinone (TQ) and Ferulic Acid (FA) 25 μM TQ + 250 μM FA, 50 μM TQ + 350 μM FA, 50 μM TQ + 450 μM FA, 100 μM TQ + 350 μM FA, 100 μM TQ + 450 μM FA)	[79]
	MCF7 and 4T1	Induces apoptosis	↓ viability, structural changes in cancer cells as compared to normal cells, ↑ apoptosis, ↑ lipid peroxidation, ↑ mitochondrial damage, ↑ cell death	FA-Nanosponges 5, 10, 20, 40, 80, 125, 250, 500, 750, and 1000 µM	[80]
	MDA-MB-231	-	↓ S phase, ↑ antiproliferative effects, ↑ sensitivity to UV treatment	0–10 µM	[81]
	MDA-MB-231	Induces apoptosis and inhibits metastasis	↓ viability, ↑ apoptosis, ↓ metastatic potential, reversal of epithelial-mesenchymal transition (EMT), ↑ caspase-3, ↓ migration across the wound edges, ↓ migration, ↓ vimentin, ↑ E-cadherin	3, 10, 30 and 100 µM	[49]
	MCF-7, MDAMB-231 and HS578T	Induces apoptosis	↓ proliferation, ↑ cytotoxicity, ↑ p53, ↑ Bax, ↑ caspase-9	0–75 μM	[82]
Lung	A549	Induces apoptosis	↓ proliferation, ↓ oxidative stress, ↓ GSH, ↑ Keap1, ↓ Nrf2 nuclear level, ↑ apoptotic population, ↓ p-p38 MAPK level, ↓ activation of Akt/MAPK, ↓ p-STAT3, ↓ Cox-2, ↓ MMP-9 and VEGF, ↓ PECAM1, ↑ arrest at at G2/M phase, ↑ p53 and p21 protein, ↓ Cdc25C, ↑ active caspase 9,3, ↑ Bax, ↓ Bcl-2, ↑ radiation sensitivity	ferulic acid −10–400 μM, Gamma radiation 5, 7.5, 10 and 15 Gy (60 Co)	[83]
	A549	Inhibits metastasis	↓ Proliferation, ↑ G0/G1 phase (cell cycle arrest), ↓ migration and invasion, ↓ Bcl-2, ↑ Bax, ↑ Bax/Bcl-2 ratio, ↓ MMP-2 and MMP-9, ↓p- ERK and p-p38, it increased JNK expression, ↓ p-AKT, p-mTOR, p-MEK, and p-ERK	Ferulic acid derivative FXS-3 0.2–50 µM	[84]
Hepatocellular	HepG2	Induces apoptosis	↓ proliferation, ↓ oxidative stress, ↓ GSH, ↑ Keap1, ↓ Nrf2 nuclear level, ↑ apoptotic population, ↓ p-p38 MAPK level, ↓ activation of Akt/MAPK, ↓ p-STAT3, ↓ Cox-2, ↓ MMP-9 and VEGF, ↓ PECAM1, ↑ arrest at at G2/M phase, ↑ p53 and p21 protein, ↓ Cdc25C, ↑ active caspase 9,3, ↑ Bax, ↓ Bcl-2, ↑ radiation sensitivity	ferulic acid -10–400 μM, Gamma radiation 5, 7.5, 10 and 15 Gy (60 Co)	[83]
	Huh-7 and HepG2	Induces apoptosis	↓ viability, ↑ structural changes, ↑ROS, ↓ MMP, ↑ DNA damage, ↓ percent of cells in G0/G and G2/M, ↑ S phase, ↑ γH2AX, ↑ Bax, Bad, cleaved caspase 3	ZnONPs with ferulic acid (ZnONPs-FAC) 0.05, 0.1, 1, 5, 10 and 20 µg/ml	[85]
Pancreatic	MIA PaCa-2	Induces apoptosis	↓ cell viability and colony formation, ↑ p53, Bax, PTEN caspase 3 and 9	150 μM, 200 μM, 300 μM, 400 μM, 500 μM, 750 μM and 1 mM	[86]
Cervical	HeLa and Caski	Induces apoptosis	↓ viability, ↑ DNA condensation, ↑ apoptosis, ↑ pro-caspase-3, pro-caspase-8, pro-caspase-9 and PARP, ↓ Bcl-2 and Mcl-1, ↑ Bax and ROS, ↓p-Akt and p-PI3K	4–20 µM	[28]
	Hela and Caski	Induction of cell cycle arrest and autophagy	↓ invasion, ↓ MMP-9, ↑ arrest in G0/G1 phase, ↑ p53 and p21, ↓ Cyclin D1 and Cyclin E, ↓ LC3-II, Beclin1 and Atg12-Atg5	0, 0.5, 1.0,1.5 and 2.0 mM	[32]
	HeLa	-	↓ Cell viability	ferulic acid nanohybrids 1, 5, 10, 20, 30, 40, and 50 μM	[87]
	HeLa and ME-180	Enhances radiation effects by increasing lipid peroxidative markers	↓ viability ↓ GSH, ↑ TBARS, CD and LHO, ↓ SOD, CAT and GPx, ↑ DNA damage, ↑ intracellular ROS levels (results by ferulic acid + irradiation in comparison with radiation or ferulic acid treatment alone)	ferulic acid (1, 5, 10, 20, 30 and 40 µg/mL) + radiation (2, 4, 6, 8, 10, 12 and 15 Gy)	[88]
Prostate	PC-3	Induces apoptosis	↓ proliferation, ↑ ATR, ATM, CDKN1A, CDKN1B, E2F4, RB1, and TP53 (Gene expression), ↓ CCND1, CCND2, CCND3, CDK2, CDK4, and CDK6 (gene expression) ↓ CDK4 and BCL2 (protein expression), ↓ invasion and colony formation	20, 30, 50, 75, 100, 150, 200, 250, 300, 350, 500, 750, 900 µM, 1, 2 mM	[25]
	LNCaP	Induces apoptosis	↓ proliferation, ↑ CASP1, CASP2, CASP8, CYCS, FAS, FASLG, and TRADD (gene expression), ↓ BCL2 and XIAP (gene expression), ↓CDK4 and BCL2 (protein expression), ↓ invasion and colony formation	20, 30, 50, 75, 100, 150, 200, 250, 300, 350, 500, 750, 900, 1000 and 2000 µM	[25]
Colorectal	HCT- 116 and HT-29	Induces apoptosis	↑ antiproliferative effects, ↑ arrest at the G1 phase, ↓ S phase, ↑ Early apoptotic cells, ↑ Caspase 3, 8, and 9 activity	0,0.25,0.5,1.0 and 1.5 mM	[89]
	HCT116	Induces apoptosis	↓ proliferation, ↑ p15 (mRNA level)	Ferulic acid-bound resveratrol- 0, 0.625, 1.25, 2.5, 5, 10 and 20 µM	[90]

**Table 2:** Representation of anticancer activities of ferulic acid in vivo models.

Type of Cancer	Animal Models	Effects	Mechanisms	Dosage	Duration	References
Melanoma	Female C57BL/6 mice xenografted with B16F10 cells	Inhibited tumor angiogenesis	↓ tumor volume and weight, ↓ p-FGFR1Y1 positive cells, ↓ FGFR1, ↓p-Akt, ↓ p-PI3K	0, 10, 30, 50 mg/kg	30 days	[91]
Sarcoma	ICR male mice transplanted with S180 cells	-	↑ diosbulbin B-induced anti-tumor activity	ferulic acid 8 mg/kg + DB 32 mg/kg	-	[92]
Colon	Male BALB/c mice xenografted with CT 26 cells	Inhibited tumor growth	↑ tumor regression, ↑ cleaved caspase 3, ↑ tumor shrinkage, ↑ damage in tumor cell parenchyma, ↑ shrinkage in tissues, ↑ nuclear fragmentation, ↑ apoptotic body formation at the neoplastic region	ferulic acid 50 mg/kg + 2 Gy dose of radiation	27 days	[86]
Breast	Female BALB/c nude xenografted with MDA-MB-231	Inhibited tumor metastasis	↓ toxicity, ↓ tumor volumes and weights, ↓ proliferation (Ki67 staining), ↑ apoptosis (active caspase-3 staining), ↓ tumor nodules on the surface of the lungs and liver	100 mg/kg	28 days	[84]
Lung	C57BL/6 mice transplanted with A549 cells	Inhibited tumor metastasis	↓ tumor volume, ↓ pulmonary metastatic nodules, ↓ pulmonary tumor metastasis	FXS-3 at 25–100 mg/kg	27 days	[93]
Hepatocellular	Wistar albino rat	Inhibited tumor metastasis	↓ nodular formation, ↓ GST-P + ive, ↓ Ki67 and 8-OHdG positivity, ↓ ALT, AST, ALP, γ-GT and TBARS (liver marker enzymes)	ZnONPs with ferulic acid (ZnONPs-FAC) 3.6 µg/mL µg/ml	-	[88]
Pancreatic	SCID mice	Inhibited tumor growth ↓↑	↓ tumor volume, ↓ PCNA and MKI67, and ↑ p-RB, ↑ p21, ↑ p-ERK1/2	75 mg/kg	35 days	[94]

## Data Availability

Not applicable.
